# Socioeconomic Vulnerability and Its Associations With Dietary Patterns and Obesity Degree Among Children in Families Across Six European Countries: The Feel4Diabetes‐Study

**DOI:** 10.1111/ijpo.70072

**Published:** 2026-01-20

**Authors:** Lubna Mahmood, Luis A. Moreno, Peter Schwarz, Lieven Annemans, Greet Cardon, Soukaina Hilal, Imre Rurik, Violeta Iotova, Natalya Usheva, Tsvetalina Tankova, Costas Anastasiou, Yannis Manios, Esther M. Gonzalez‐Gil, the Feel4Diabetes‐Study Group, Jaana Lindström, Konstantinos Makrilakis, Winne Ko, Kalliopi Karatzi, Odysseas Androutsos, George Moschonis, Spyridon Kanellakis, Christina Mavrogianni, Konstantina Tsoutsoulopoulou, Christina Katsarou, Eva Karaglani, Irini Qira, Efstathios Skoufas, Konstantina Maragkopoulou, Antigone Tsiafitsa, Irini Sotiropoulou, Michalis Tsolakos, Effie Argyri, Mary Nikolaou, Eleni‐Anna Vampouli, Christina Filippou, Kyriaki Apergi, Amalia Filippou, Gatsiou Katerina, Efstratios Dimitriadis, Jaana Lindström, Tiina Laatikainen, Katja Wikström, Jemina Kivelä, Päivi Valve, Esko Levälahti, Eeva Virtanen, Tiina Pennanen, Seija Olli, Karoliina Nelimarkka, Vicky Van Stappen, Nele Huys, Ruben Willems, Samyah Shadid, Patrick Timpel, Konstantinos Makrilakis, Stavros Liatis, George Dafoulas, Christina‐Paulina Lambrinou, Angeliki Giannopoulou, Winne Ko, Ernest Karuranga, Luis Moreno, Fernando Civeira, Gloria Bueno, Pilar De Miguel‐Etayo, María L. Miguel‐Berges, Natalia Giménez‐Legarre, Paloma Flores‐Barrantes, Aleli M. Ayala‐Marín, Miguel Seral‐Cortés, Lucia Baila‐Rueda, Ana Cenarro, Estíbaliz Jarauta, Rocío Mateo‐Gallego, Natalia Usheva, Kaloyan Tsochev, Nevena Chakarova, Sonya Galcheva, Rumyana Dimova, Yana Bocheva, Zhaneta Radkova, Vanya Marinova, Yuliya Bazdarska, Tanya Stefanova, Timea Ungvari, Zoltán Jancsó, Anna Nánási, László Kolozsvári, Csilla Semánova, Éva Bíró, Emese Antal, Sándorné Radó, Remberto Martinez, Marcos Tong

**Affiliations:** ^1^ Growth, Exercise, Nutrition and Development (GENUD) Research Group University of Zaragoza Zaragoza Spain; ^2^ Instituto Agroalimentario de Aragón (IA2) Zaragoza Spain; ^3^ Instituto de Investigación Sanitaria de Aragón (IIS Aragón) Zaragoza Spain; ^4^ Department for Prevention and Care of Diabetes Medical Faculty Carl Gustav Carus at the Technical University of Dresden Dresden Germany; ^5^ Paul Langerhans Institute Dresden of the Helmholtz Center Munich at University Hospital and Faculty of Medicine, TU Dresden Dresden Germany; ^6^ German Center for Diabetes Research (DZD e.V.) Neuherberg Germany; ^7^ Department of Public Health and Primary Care Ghent University Gent Belgium; ^8^ Department of Movement and Sports Sciences Ghent University Ghent Belgium; ^9^ Doctoral School of Health Sciences University of Debrecen Debrecen Hungary; ^10^ Department of Family Medicine Semmelweis University Budapest Hungary; ^11^ Department of Pediatrics Medical University of Varna Varna Bulgaria; ^12^ Division of Diabetology, Department of Endocrinology Medical University of Sofia Sofia Bulgaria; ^13^ Department of Nutrition and Dietetics, School of Health Science & Education Harokopio University Athens Greece; ^14^ Institute of Agri‐Food and Life Sciences Hellenic Mediterranean University Research Centre Heraklion Greece; ^15^ Grupo de Estilos de Vida, Microbiota y Salud (GESMUD). Unidad Predepartamental de Enfermería. Facultad de Ciencias de la Salud. Universidad de La Rioja Logroño Spain

**Keywords:** children, dietary intake, Europe, obesity, SES, socioeconomic vulnerability

## Abstract

**Introduction:**

Previous studies suggest that children from lower socioeconomic status may be at higher risk of unhealthy eating. This study aims to examine the cumulative socioeconomic vulnerabilities and their association with dietary patterns and obesity levels in European children.

**Methods:**

A total of 9664 parent‐child dyads (79% mothers; 50.8% girls) from six European countries participated in the study. Families provided baseline information on energy balance‐related behaviours and socioeconomic factors through questionnaires. Children's dietary habits were evaluated using semi‐quantitative food frequency questionnaires, and anthropometric data were collected. Vulnerabilities were quantified through a composition of variables based on household income, parental education and employment. Multivariable and logistic regression analyses were performed.

**Results:**

The study found significant associations between socioeconomic vulnerability and children's dietary habits and body mass index. Higher vulnerability was linked to a lower probability of fruit and vegetable intake, and a higher probability of consuming red meat, and energy‐dense snacks. Children with the highest vulnerability had nearly four times higher odds of overweight/obesity (OR = 3.82, 95% CI: 3.05–4.76).

**Conclusions:**

The findings of this study indicate that European children from families with a high socioeconomic vulnerability tend to have an unhealthy dietary profile and a higher degree of obesity. Therefore, public health programs should prioritise families facing high socioeconomic vulnerabilities promoting healthy eating to prevent childhood obesity at early stages.

**Trial Registration:**

Clinical trials registry http://clinicaltrials.gov: NCT02393872

## 1 Introduction

Socioeconomic vulnerabilities represent a significant economic burden, contributing to healthcare costs [1]. In Europe, for instance, health inequalities cost an estimated €980 billion annually, equivalent to 9.4% of the Gross Domestic Product [1]. The link between socioeconomic status (SES), diet quality, and obesity or non‐communicable diseases such as Type 2 diabetes and cardiovascular disease, is well documented [2]. Specifically, studies in high‐income countries consistently show that SES is a major determinant of dietary habits and obesity, with individuals from lower‐income backgrounds more likely to consume energy‐dense, nutrient‐poor foods, while those from higher‐income backgrounds tend to adopt healthier diets [2, 3]. This disparity arises largely from the lack of nutritional education and the lower cost of energy‐dense foods, contributing to poorer dietary quality and higher obesity prevalence among lower‐SES groups [2, 4].

Socioeconomic vulnerabilities have a profound impact as childhood socioeconomic disadvantage has lasting health effects into adulthood [4, 5]. Children from lower‐SES families often consume poorer diets, with lower fruit and vegetable intake [5] while they have a high intake of snack foods, fast foods, and sweetened beverages [6]. These dietary patterns characterised by consumption of high‐fat and high‐energy density foods [6], elevate obesity rates, and heighten cardiovascular disease risk in adulthood [7].

However, a single‐dimensional measure of socioeconomic vulnerability, such as income, education, or occupation, fails to capture the complexities influencing nutrition and health in marginalised settings like urban slums [8]. To address these challenges, it is essential to develop a comprehensive framework that integrates health, nutritional, and socioeconomic indicators. Such a framework can help identify and address disparities, enabling targeted support for vulnerable populations [9].

Early socioeconomic conditions, particularly those influencing diet, have a significant impact on long‐term health, highlighting the importance of tackling disparities to prevent chronic diseases [10]. Thus, understanding which SES indicators—commonly education, social class, or income—most strongly associate with diet is crucial for tackling health inequalities [11, 12, 13, 14]. Therefore, this study fills the gap by investigating cumulative socioeconomic vulnerabilities, using a combination of variables including household income, parental education and employment, and their association with dietary patterns and obesity in European children. These findings aim to enhance understanding of how socioeconomic vulnerabilities are associated with childhood health and to inform strategies addressing diet‐related public health challenges.

## 2 Methods

### 2.1 Study Design

The Feel4Diabetes study aimed to prevent obesity and related comorbidities by promoting healthy lifestyles among 11 396 families across six European countries. The countries were categorised into three groups: high‐income countries (Belgium and Finland), countries under austerity measures following the economic crisis (Greece and Spain), and low‐income countries (Bulgaria and Hungary). Children in the first three grades of primary school, along with their parents, were invited to participate in the study, which was conducted between 2016 and 2018. Data collection occurred at baseline in 2016, with follow‐up assessments in 2017 and 2018. The study protocol has been previously published [15]. The Feel4Diabetes study is registered in the clinical trials registry (http://clinicaltrials.gov, NCT02393872).

The study adhered to the Declaration of Helsinki and the Council of Europe conventions on human rights and biomedicine. Ethical clearance was obtained from the relevant committees and authorities in each participating country: Belgium (ethical approval code: B670201524237), Finland (174/1801/2015), Greece (46/3‐4‐2015), Spain (CP03/2016), Hungary (20 095/2016/EKU), and Bulgaria (52/10‐3‐2015). Written informed consent was obtained from all participants prior to enrollment in the study.

### 2.2 Study Sample

The current study utilised the baseline cross‐sectional data from theFeel4Diabetes study that included 11 396 families. Parents provided information about their children through completed questionnaires, along with anthropometric measurements. To avoid duplicating parental data, only one child per family was randomly selected when families had more than one child, and this child was linked to the reported parental information. For the current analysis, only dyads with complete data were considered, resulting in a final sample of 9664 out of the 11 396 eligible children.

### 2.3 Socioeconomic Status Assessment

In the Feel4Diabetes project, sociodemographic information was collected using standardised, validated questionnaires specifically developed for the study. Data included the child's age and sex, as well as parental education, occupational status, and household income security. Parental education was originally categorised into six levels (less than 6 years, 7–9 years, 10–12 years, 13–14 years, 15–16 years, and more than 16 years of schooling) but was dichotomized into ≤ 12 years (up to the end of secondary education) versus > 12 years for analysis. Employment status was assessed with six response options (homemaker, full‐time employment, part‐time employment, unemployed, student, or retired) and was subsequently classified into unemployed (homemakers and unemployed) versus employed (full‐time, part‐time, students, or retirees). Household income security was measured through a qualitative question on how easily parents managed household expenses, with answers recorded on a six‐point Likert scale ranging from very difficult to very easy, and later recoded into difficult versus easy. Maternal and paternal education and occupation were considered separately, while income security was assessed at the family level.

The SES vulnerability indicators were defined as the five dichotomized factors: maternal and paternal education, maternal and paternal occupation, and household income security. For each indicator, a value of 1 was assigned if the vulnerability criterion was met, and 0 if not. For example, if either parent had ≤ 12 years of education, the child received 1 point for that indicator, and if both parents had > 12 years, this contributed 0 points. The same principle was applied separately for maternal and paternal occupation. The total number of vulnerabilities was then used to create SES vulnerability categories, ranging from 0 (no vulnerabilities) to 4 (maximum accumulation of disadvantages). In theory, the sum of indicators could have ranged from 0 to 5, but in this sample the maximum observed value was 4; therefore, categories were restricted to 0–4 for the analyses. SES data were available for 9664 families.

### 2.4 Dietary Assessment

A semi‐quantitative food frequency and eating behaviour questionnaire was provided to families, that one parent completed at home [16]. The questionnaire gathered information on the children's frequency of consuming breakfast, grains, fresh fruits, vegetables, legumes, red meat, poultry, fish and seafood, dairy products, savoury snacks (e.g., croissants and cheese pie), sweets (e.g., pancakes, cookies, or chocolates), and soft drinks. Breakfast consumption was assessed with the question: ‘On how many days does your child usually eat breakfast?’ and parents could select from the following options: never, less than 1 time/week, 1–2 times/week, 3–4 times/week, 5–6 times/week, or daily.

For the dietary intake of other food items, parents were asked: ‘How many servings of (item) does your child eat?’ They could then choose from the following options: ‘One or less than one serving per week’, ‘2 servings per week’, ‘3‐4 servings per week’, ‘5‐6 servings per week’, ‘1‐2 servings per day’, ‘3‐4 servings per day’, or ‘5 or more servings per day’. The portion size for each food item was defined using household units. For example, one serving of fresh fruit was equivalent to a medium‐sized fruit (e.g., one apple = 90 g), two small fruits (e.g., apricots), or half a cup of chopped fruit or berries. Responses ranged from ‘less than one serving per week’ to ‘5 or more servings per day’. In this study, the consumption of each food item was converted to daily intake in grams through multiplying the number of servings consumed by the standard portion size.

### 2.5 Anthropometric Measurements

Children's height and weight were measured at schools by trained researchers following a standardised procedure [13]. Bodyweight was recorded to the nearest 0.1 kg using a Seca 813 digital flat scale, with children barefoot and wearing light clothing. Height was measured to the nearest 0.1 cm using a Seca 217 stadiometer, with the head positioned in the Frankfurt plane. Two readings were taken for both height and weight, and the average of these values was used for analysis. If the two measurements differed by more than 100 g for weight or more than 1 cm for height, a third measurement was taken. Body mass index (BMI) was calculated as weight in kilograms divided by height in meters squared (kg/m^2^). Based on the BMI values, children were categorised into three groups: underweight, normal weight, and overweight/obesity. Gender‐ and age‐specific BMI z‐scores (zBMI) were calculated following the method outlined by Cole et al. [17].

### 2.6 Statistical Analysis

Descriptive data on participants' characteristics are presented as percentages or means for categorical or continuous variables, respectively. Kolmogorov–Smirnov test was used to check the distribution of the included variables. Since gender interactions were observed in the associations between dietary habits and weight status among children, all analyses were stratified by gender. Student's *t*‐tests were used to compare means of continuous variables by gender, and Pearson's chi‐square test was used in the case of categorical variables. We reported these results stratified by country, and BMI categories.

For analytical purposes, education level, occupation, and household income security were each dichotomized into two categories. Education levels were grouped as ≤ 12 years versus > 12 years, where 12 years indicates completion of primary and secondary education. Occupation categories were consolidated into unemployed (including stay‐at‐home and unemployed individuals) versus employed (encompassing those working full‐time, part‐time, students, and retirees). Similarly, household income security, initially assessed using six Likert scale options ranging from ‘very difficult’ to ‘very easy’, was dichotomized into two categories: difficult and easy. Moreover, BMI categories were classified into normal weight versus overweight/obesity. Multivariable regression analysis was used to examine the association between socioeconomic vulnerability components and the intake of children. The regression analysis was adjusted for children's age, sex, country, and BMI category as well as parental gender, age, marital status, group (control vs. intervention) and BMI. The association between socioeconomic vulnerability and dietary intake of children from various food groups was analysed, using a logistic regression model. Binary logistic regression was performed to explore the associations between SE vulnerability and BMI, adjusted for children's age, sex, country, and parental gender, age, marital status, BMI and group (control vs. intervention). Individual SES indicators and the combination of variables were not included in the same model. Instead, analyses were run separately to avoid multicollinearity.

Statistical analyses were carried out using IBM‐SPSS (Version 26.0. Armonk, NY: IBM Corp, USA), with a *p* < 0.05 representing statistical significance for all tests.

## 3 Results

A total of 9664 parents–child dyads were included in the analysis. Table 1 presents descriptive statistics for parents and children, highlighting key demographic and socioeconomic characteristics. The average age of parents is 37.6 years, while children average 8.14 years. A notable gender disparity exists, with 79.0% of parents identifying as women and 50.8% of the children being girls. Education levels reveal that 57.2% of parents have more than 12 years of education. Regarding employment status 68.8% of parents are employed, and 72.3% are married, suggesting supportive family structures. In terms of household income security, 51.0% of parents report having easy access to financial resources.

**TABLE 1:** Descriptive statistics.

Characteristics	Parents	Children
Age (in years)	37.6 (4.3)	8.14 (0.9)
Sex (% women)	79.0%	50.8%
Education level (> 12 years of education)	57.2%	—
Employment status (employed)	68.8%	—
Marital status (married)	72.3%	—
Household income security (easy)	51.0%	—
Socioeconomic vulnerability		
Category 0	14.42%	—
Category 1	20.08%	—
Category 2	22.42%	—
Category 3	24.83%	—
Category 4	18.25%	—
Country of residence		
Belgium	14.6%	—
Finland	12.5%	—
Spain	13.7%	—
Greece	18.5%	—
Hungary	13.8%	—
Bulgaria	26.9%	—
BMI^1^ (kg/m^2^)		
Normal	74.3%	72.1%
Overweight/obesity	25.7%	27.9%
BMI z‐score	—	0.69 (1.1)

*Note:* *N* = 9664 parents and children. This table provides mean (SD) for the continuous variables and frequency (%) for the categorical variables. Category 0 = no SES vulnerability, Category 1 = very low SES vulnerability, Category 2 = low vulnerability, Category 3 = moderate SES vulnerability, Category 4 = high SES vulnerability. 1 BMI: Body Mass Index. BMI z‐scores were calculated according to Cole et al.

The socioeconomic vulnerability combination of variables indicates that 14.42% of families fall into SES category 0, with categories 1 through 4 at 20.08%, 22.42%, 24.83%, and 18.25%, respectively, reflecting a higher proportion experiencing moderate to high socioeconomic vulnerability. Geographically, the selected participants were most highly represented among parents in Bulgaria (26.9%), followed by Greece (18.5%) and Spain (13.7%). This variation may reflect differing economic conditions across countries. Regarding BMI, 74.3% of parents and 72.1% of children are in the normal weight range, while 25.7% of parents and 27.9% of children are classified as having overweight or obesity.

### 3.1 Association Between Socioeconomic Vulnerability and Children's Dietary Intake

Table 2 provides insights into the association between socioeconomic vulnerability categories (ranging from 0 to 4) and children's dietary intake, categorised by frequency of consumption.The analysis found that higher socioeconomic vulnerability categories were associated with increased odds of red meat consumption among children, with the association reaching statistical significance in the more vulnerable groups (e.g., SES category 4: OR = 1.25, 95% CI: 1.05–1.50, *p* < 0.05). A similar, though less pronounced, pattern was observed for white meat and poultry intake, with slightly higher odds in higher SES vulnerability groups; however, these associations did not reach statistical significance. Fish and seafood intake showed minimal variation across SES levels, and no significant associations were detected.

**TABLE 2:** Association between Socioeconomic vulnerability and children's dietary intake.

Food intake (daily)	OR (95% CI)
Milk and milk products	
SES vulnerability category 0	Reference
SES vulnerability category 1	1.10 (0.95–1.28)
SES vulnerability category 2	**1.25 (1.05–1.48)**
SES vulnerability category 3	**1.35 (1.12–1.63)**
SES vulnerability category 4	**1.45 (1.20–1.76)**
Grain bread and BF cereals	
SES vulnerability category 0	Reference
SES vulnerability category 1	1.12 (0.96–1.31)
SES vulnerability category 2	**1.28 (1.05–1.55)**
SES vulnerability category 3	**1.50 (1.22–1.84)**
SES vulnerability category 4	**1.65 (1.34–2.03)**
Breakfast consumption	
SES vulnerability category 0	Reference
SES vulnerability category 1	0.88 (0.75–1.04)
SES vulnerability category 2	**0.78 (0.65–0.92)**
SES vulnerability category 3	**0.65 (0.54–0.79)**
SES vulnerability category 4	**0.55 (0.45–0.67)**
Fruits	
SES vulnerability category 0	Reference
SES vulnerability category 1	0.93 (0.80–1.08)
SES vulnerability category 2	0.85 (0.72–1.01)
SES vulnerability category 3	**0.70 (0.58–0.84)**
SES vulnerability category 4	**0.60 (0.49–0.73)**
Vegetables	
SES vulnerability category 0	Reference
SES vulnerability category 1	0.90 (0.76–1.06)
SES vulnerability category 2	**0.78 (0.64–0.94)**
SES vulnerability category 3	**0.65 (0.52–0.80)**
SES vulnerability category 4	**0.55 (0.44–0.69)**
Legumes	
SES vulnerability category 0	Reference
SES vulnerability category 1	1.08 (0.92–1.28)
SES vulnerability category 2	**1.25 (1.03–1.52)**
SES vulnerability category 3	**1.42 (1.15–1.75)**
SES vulnerability category 4	**1.60 (1.30–1.98)**
Red meat	
SES vulnerability category 0	Reference
SES vulnerability category 1	1.05 (0.92–1.21)
SES vulnerability category 2	1.12 (0.96–1.31)
SES vulnerability category 3	**1.20 (1.02–1.42)**
SES vulnerability category 4	**1.28 (1.08–1.53)**
White meat and poultry	
SES vulnerability category 0	Reference
SES vulnerability category 1	1.00 (0.85–1.17)
SES vulnerability category 2	1.05 (0.89–1.24)
SES vulnerability category 3	1.12 (0.95–1.32)
SES vulnerability category 4	1.18 (0.99–1.40)
Fish and seafood	
SES vulnerability category 0	Reference
SES vulnerability category 1	1.03 (0.88–1.22)
SES vulnerability category 2	1.08 (0.91–1.29)
SES vulnerability category 3	1.15 (0.96–1.39)
SES vulnerability category 4	1.20 (1.00–1.45)
Salty snacks	
SES vulnerability category 0	Reference
SES vulnerability category 1	**1.40 (1.15–1.70)**
SES vulnerability category 2	**1.60 (1.30–1.98)**
SES vulnerability category 3	**1.95 (1.60–2.40)**
SES vulnerability category 4	**2.25 (1.85–2.75)**
Sweets	
SES vulnerability category 0	Reference
SES vulnerability category 1	**1.30 (1.05–1.60)**
SES vulnerability category 2	**1.55 (1.25–1.90)**
SES vulnerability category 3	**1.90 (1.55–2.33)**
SES vulnerability category 4	**2.25 (1.85–2.75)**
Sugar‐sweetened beverages	
SES vulnerability category 0	Reference
SES vulnerability category 1	**1.35 (1.10–1.65)**
SES vulnerability category 2	**1.60 (1.30–1.98)**
SES vulnerability category 3	**1.95 (1.60–2.38)**
SES vulnerability category 4	**2.35 (1.95–2.85)**

*Note:* *N* = 9664 parents and children. The regression analysis was adjusted for children's age, sex, country, and parental gender, age, marital status, group (control vs. intervention), and BMI. Odds Ratio. CI: Confidence Interval. *p* < 0.05 (Bold indicate significance). Breakfast consumption was categorised as [less than 5 days/week, 5 or more days/week]. Food consumption was categorised as [less than 3 times/day, 3 or more times/day]. Socioeconomic vulnerability combination of variables (0–4) ranged from best to worse. The reference category is ‘0’. BMI categories from better to worse (normal weight, overweight/obesity).

### 3.2 Association Between Socioeconomic Vulnerability Components and Children's Dietary Intake

Our analysis revealed that among the three examined factors—parental education, household income, and employment—parental education was significantly associated with the intake of the majority of food groups consumed by children (Table 3). Higher education levels were associated with increased consumption of healthier foods, such as milk (*β* = 0.32, *p* = 0.001), legumes (*β* = 0.25, *p* = 0.005), and fruits (*β* = 0.15, *p* = 0.025), while there was a lower intake of sweets (*β* = −0.20, *p* = 0.005) and sugar‐sweetened beverages (*β* = −0.25, *p* = 0.001). Employment showed weaker associations with food intake, with small positive effects on breakfast consumption (*β* = 0.12, *p* = 0.420) and milk (*β* = 0.10, *p* = 0.250). Household income security was linked to both healthier foods, like milk (*β* = 0.08, *p* = 0.040), and unhealthier choices, including sweets (*β* = 0.04, *p* = 0.020). In summary, parental education emerged as the factor associated with the majority of healthier dietary patterns in children, while employment and income security showed more mixed results.

**TABLE 3:** Association between Socioeconomic vulnerability components and children's dietary intake.

	Socioeconomic vulnerability components					
	Education		Employment		Household income security	
	*β*	*p*	*β*	*p*	*β*	*p*
Breakfast consumption (times/week)^a^	0.18	**0.010**	0.12	0.420	−0.05	**0.020**
Children food consumption (g/day)						
Milk and milk products^b^	0.32	**0.001**	0.10	0.250	0.08	**0.040**
Grain bread and BF cereals^c^	0.20	**0.003**	0.08	0.350	−0.06	0.050
Fruits	0.15	**0.025**	0.05	**0.030**	−0.12	0.100
Vegetables	0.10	**0.010**	0.07	0.180	−0.10	0.060
Legumes	0.25	**0.005**	0.12	0.800	0.02	**0.030**
Red meat	0.12	0.090	0.03	0.230	0.05	0.400
White meat and poultry	0.08	0.150	0.06	0.420	0.03	0.220
Fish and seafood	0.10	0.080	0.05	0.590	0.02	0.300
Salty snacks	0.18	**0.012**	0.10	0.340	0.05	0.050
Sweets	−0.20	**0.005**	0.11	0.420	0.04	**0.020**
Sugar sweetened‐beverages	−0.25	**0.001**	0.08	0.300	0.06	**0.040**

*Note:* *N* = 9664 parents and children. *p* < 0.05 (Bold indicate significance). Regression analyses were adjusted children's age, sex, country, and BMI category as well as parental gender, age, marital status, group (control vs. intervention), and BMI. BF: breakfast; β: Standardised coefficient.

^a^ Breakfast consumption was categorised as [1 day or less/week, 2–4 days/week, 5 or more days/week].

^b^ Cheese was not counted.

^c^ Rice and pasta were not mentioned under grains group in the questionnaire.

### 3.3 Association Between Socioeconomic Vulnerability and Children's BMI

Table 4 outlines the association between socioeconomic vulnerability categories and children's body mass index (BMI), specifically contrasting normal weight against overweight/obesity, as determined by logistic regression. The reference category is represented by children with a socioeconomic vulnerability category of 0. For children with a socioeconomic vulnerability category of 1, the odds ratio (OR) is 1.25 (95% CI: 0.95, 1.43), suggesting a higher likelihood of being in the overweight/obesity category compared to the reference group, although these findings approach significance without reaching it. Children in Category 2 exhibit a more pronounced association, with an OR of 1.63 (95% CI: 1.21, 2.18), indicating a significant increase in odds for being in the overweight/obesity category. The results become even more compelling for children with a socioeconomic vulnerability Category of 3, which shows an OR of 2.25 (95% CI: 1.79, 2.87), reflecting a substantial increase in the likelihood of overweight or obesity. Finally, children with the highest socioeconomic vulnerability category of 4 demonstrate a striking OR of 3.82 (95% CI: 3.05, 4.76), indicating that they are nearly four times more likely to be classified as having overweight/obesity compared to those in the reference group.

**TABLE 4:** Association between Socioeconomic vulnerability and children's BMI.

Socioeconomic vulnerability score	BMI of children (normal weight vs. overweight/obesity)	
	OR	(95% CI)
Category 0	Ref.	Ref
Category 1	1.25	(0.95, 1.43)
Category 2	**1.63**	**(1.21, 2.18)**
Category 3	**2.25**	**(1.79, 2.87)**
Category 4	**3.82**	**(3.05, 4.76)**

*Note:* *N* = 9664 parents and children. The regression analysis was adjusted for children's age, sex, country, and parental gender, age, marital status, group (control vs. intervention), and BMI. Odds Ratio. CI: Confidence Interval. BMI: Body Mass Index. *p* < 0.05 (Bold indicate significance). The reference category is ‘0’. BMI categories from better to worse (normal weight, overweight/obesity).

## 4 Discussion

This study, part of the Feel4Diabetes project, examines the dietary habits of children from socioeconomically vulnerable families across six European countries. While the relationship between socioeconomic vulnerability and dietary patterns has been frequently explored, this study is among the first to assess it using a combination of variables approach among European children.

Results show higher vulnerability correlates with increased intake of dairy, grains, energy‐dense snacks, and breakfast frequency, alongside reduced fruit and vegetable consumption, which is further associated with greater overweight and obesity prevalence.

Our study found that parental education was significantly associated with the intake of the majority of food groups consumed by children, with higher parental education levels associated with healthier food consumption patterns in children. Interestingly, however, regarding the prevalence of socioeconomic vulnerability components, household income security followed by unemployment was the most commonly achieved aspects. Parental education was the least common, even if it was the indicator most associated with diet. These results are consistent with previous studies [18, 19], which also highlight the strong influence of parental education on children's dietary habits.

Socioeconomic vulnerability is essential for evaluating children's dietary patterns and obesity, incorporating factors such as parents' income, education, and occupation. The combination of variables influences access to nutritious food and health. Various vulnerability indices, like the Social Vulnerability Index [20], Livelihood Vulnerability Index [21], have shown that higher scores are linked to increased fast food intake, higher obesity risk, and reduced exercise opportunities [18]. However, many of these indices were inapplicable to our study due to unavailable data, such as precise income levels, water access, and metrics on health deprivations, living environments, climate change, and disaster resilience. Instead, this study used a socioeconomic vulnerability combination of variables specifically developed for Feel4Diabetes participants, previously employed to examine cumulative vulnerabilities and differences in children's food intake [22].

Our results confirmed that children from socioeconomically vulnerable backgrounds may have a lower frequency of breakfast consumption. Data from 41 countries in the HBSC study showed reduced daily breakfast consumption among children with lower socio‐demographic status, influenced by socioeconomic conditions, cultural norms, and school breakfast program availability [23]. Similarly, skipping breakfast was more prevalent among Norwegian adolescents with lower socioeconomic status [24]. Financial constraints and limited access to nutritious food or school breakfast programs contribute to this trend [25]. These factors result in inadequate breakfast consumption, negatively affecting children's nutritional intake and health. Our study also noted lower consumption of milk, dairy products, and breakfast cereals among families with high socioeconomic vulnerability, aligning with previous research [24, 25, 26]. This pattern may stem from factors such as limited income, dietary preferences, and health beliefs within this demographic [26].

In line with previous research [22, 27], this study found that families with higher socioeconomic vulnerability were more likely to consume fewer fresh and nutrient‐dense foods, such as FV. This trend is likely due to limited access to grocery stores offering fresh, nutrient‐dense food options in low‐income neighbourhoods. Families in these areas often struggle to find nutritious foods nearby, leading to fewer and often less healthy choices [28]. Additionally, a lack of nutrition education in low‐income families has been associated with a reduced emphasis on nutrient‐dense food options, with energy‐dense meals often prioritised due to time and financial constraints [29].

Our findings revealed a higher probability of red meat consumption among children with increased socioeconomic vulnerability, particularly in the most vulnerable groups, possibly due to factors such as cost, availability, or cultural preferences. However, no significant associations were observed for poultry or fish/seafood consumption, indicating that these foods do not follow the same pattern of increased probability across different levels of socioeconomic vulnerability. These results contrast with previous studies, which suggested that children from lower socioeconomic backgrounds tend to consume less red meat and other protein sources due to limited access to affordable, high‐quality foods [30].

Our findings may reflect an increased likelihood of consuming cheaper, processed red meat products, such as sausages, hot dogs, and canned meats, which are typically more affordable and readily available than healthier protein sources like fresh fish, poultry, or lean cuts of beef. These products are often high in salt, preservatives, and saturated fats, which may contribute to poorer nutritional quality. However, in the Feel4Diabetes study, the level of processing of foods was not considered so distinctions cannot be made. Previous studies have suggested that socioeconomic vulnerability can limit access to higher‐quality and more expensive protein sources, leading to either lower overall red meat consumption or a shift towards processed alternatives [31, 32]. Additionally, regional variations in food availability, market pricing, and local dietary habits, as well as cultural preferences for certain types of meat, may help explain the differences observed in our results. For example, in some areas, traditional recipes or common practices may favour processed red meat, while other regions may have easier access to fresh meats or alternative protein sources [31, 32].

Our study found that children with higher socioeconomic vulnerability scores are more likely to consume more energy‐dense food items, such as salty snacks, sweets, and sugar‐sweetened beverages. This finding aligns with previous research [30, 33]. Those studies reported that children from lower socioeconomic backgrounds tend to have diets high in processed foods and sugary drinks. A possible explanation for these findings is that families with lower SES often face financial constraints, which limit access to healthier, more expensive foods. Additionally, lower parental education levels may lead to less awareness of nutrition, further contributing to poor dietary choices. This supports the hypothesis that socioeconomic vulnerability is associated with unhealthy food consumption in children [31, 32]. These results suggest that improving socioeconomic conditions, particularly parental education and income, could help reduce unhealthy eating patterns in children.

Consistent with the findings of the IDEFICS study [34], which involved 8624 children from eight European countries, our results reveal a clear and increasing association between socioeconomic vulnerability and the likelihood of children experiencing overweight and obesity. A United States cohort study of 20 677 children found that living in low‐vulnerability neighbourhoods during early life was linked to a lower mean BMI trajectory and reduced obesity risk throughout childhood and adolescence [35]. Similarly, data from 22 European countries indicate that greater income inequality is positively associated with higher child overweight prevalence [36]. Evidence suggests that low SES correlates with elevated childhood obesity rates [37], though this pattern varies by race and SES level [38]. Moreover, children from low‐SES families often face food insecurity, limiting access to nutritious foods and fostering reliance on cheaper, calorie‐dense options high in fats and sugars [39]. Socioeconomic vulnerability also reduces access to safe recreational spaces, parks, and organised physical activities, further hindering regular exercise [40]. Additionally, financial stress can strain family dynamics, reducing time and energy for preparing healthy meals or engaging in physical activity [41]. Psychological challenges, including higher rates of depression and anxiety in economically disadvantaged families, further impede healthy lifestyles [42]. Cultural norms around body weight and food may also influence dietary choices and perceptions of obesity across socioeconomic strata [43]. These intertwined factors create a complex environment that significantly increases obesity risk among children from socioeconomically vulnerable families.

It is noteworthy to mention that the inclusion of six European countries with varying economic contexts implies substantial differences in cultural norms and policy environments that can shape children's dietary habits and obesity prevalence. For example, the IDEFICS study across eight European nations showed that adherence to a Mediterranean‐like diet varied markedly (highest in Sweden; lowest in Cyprus), and higher scores were inversely associated with overweight, obesity, and fat mass—highlighting how cultural dietary patterns influence adiposity outcomes [44]. At the policy level, systematic reviews suggest that school food environment policies—such as provision of healthy foods, regulation of competitive foods, and improvement of meal standards—can significantly increase fruit and vegetable intake and reduce consumption of sugar‐sweetened beverages, though impacts on adiposity remain modest [45]. Moreover, the heterogeneity of national school meal policies in Europe—with some countries enforcing mandatory nutrient standards and others relying on voluntary guidelines—adds to cross‐country variability in implementation and effectiveness [46]. While our study did not directly analyse these cultural and policy‐level factors, they likely contribute to the heterogeneous dietary patterns and obesity outcomes we observed across participating countries.

The present study has several limitations. First, children's data were based on parental reports, which may introduce bias. Second, self‐reported data could lead to socially desirable responses. BMI was used to assess body composition, but it may not accurately reflect body fat compared to other methods like skinfold thickness or bioimpedance analysis. The study focused on the frequency of specific meals without considering food preparation methods. Since the sample was drawn from selected regions within each country, the findings may not be generalizable to all EU member states. Additionally, the cross‐sectional design limits causal inferences. Finally, although we adjusted for multiple sociodemographic factors, the potential for residual confounding remains, as certain influential variables—such as cultural norms, children's physical activity levels, sedentary behaviours, and other lifestyle or environmental factors—were not captured in our analysis. These unmeasured factors may partially explain the observed associations. Given that missing data represented less than 5% of the sample, these omissions were considered unlikely to affect the results and were therefore excluded from the analysis. Also, since the variables included in the regression are measured in different units and on differing scales, their coefficients are not directly comparable. Thus, our findings reflect overall patterns of associations rather than direct comparison of effect sizes. However, strengths include standardised anthropometric measurements by trained researchers, a large sample from six European countries, and valuable insights into socioeconomic differences in diet quality and obesity.

In conclusion, this study showed that the dietary intake and obesity levels of European schoolchildren are significantly associated with socioeconomic vulnerability. Children from families with high socioeconomic vulnerabilities exhibited unhealthy dietary profiles and higher obesity rates, highlighting the urgent need for targeted interventions. Therefore, school‐based public health programs should prioritise families facing cumulative SES challenges. Additionally, this study emphasises the importance of incorporating community nutrition education into public health initiatives to address socioeconomic disparities effectively. Special attention must be given to parents and children from lower SES backgrounds when developing strategies for promoting healthy eating in schools and communities. The Feel4Diabetes study highlights the critical role of socioeconomic factors in shaping dietary patterns and obesity among children, advocating for comprehensive approaches in public health interventions aimed at reducing childhood overweight inequalities.

## Author Contributions

Lubna Mahmood conducted the statistical analyses, and wrote the manuscript; Yannis Manios coordinated the study; Luis A. Moreno, Yannis Manios, Peter Schwarz, Greet Cardon, Violeta Iotova, Imre Rurik, Costas Anastasiou participated in the design of the study, Esther M. Gonzalez‐Gil and Luis A. Moreno critically revised the manuscript, and supervised all procedures; Greet Cardon, Costas Anastasiou, Violeta Iotova provided essential intellectual input. All authors have read, revised and approved the final version of the manuscript.

## Ethics Statement

The study was conducted according to the guidelines of the Declaration of Helsinki. The ethical approval was obtained from the Ethical Committees of all participating European countries including Spain (ethical approval code: CP03/2016), Greece (code: 46/3‐4‐2015), Finland (code: 174/1801/2015), Belgium (code: B670201524237), Bulgaria (code: 52/10‐3‐201), and Hungary (code: 20095/2016/EKU).

## Consent

Informed consent was obtained from all participants involved in the study.

## Conflicts of Interest

The authors declare no conflicts of interest.

## Supporting information


**Data S1:** ijpo70072‐sup‐0001‐supinfo.docx.

## Data Availability

The data of the present study is available for further scientific analysis from the corresponding author on reasonable request.
